# The reemergence of visceral leishmaniasis in Brazil.

**DOI:** 10.3201/eid0202.960213

**Published:** 1996

**Authors:** J R Arias, P S Monteiro, F Zicker

**Affiliations:** Pan American Health Organization, Brasilia, Brazil.


### The Reemergence of Visceral Leishmaniasis in Brazil

Because of a complex array of factors, an increasing number of new and reemerging infectious diseases are being recognized in both industrialized and developing countries in the Americas (1,2). The expanding population, living in overcrowded conditions with inadequate housing and sanitary facilities, has been exposed to new diseases and human pathogens. For example, the appearance of the South American arenaviruses (Junin, Machupo, and Guanarito) illustrates how exploitation of new areas for human settlement and agriculture increases the likelihood that new infectious diseases will emerge. Cholera, plague, AIDS, dengue hemorrhagic fever, and urban/periurban visceral leishmaniasis are examples of new and reemerging diseases in the region.

In tropical America, zoonotic visceral leishmaniasis caused by *Leishmania chagasi*, an intracellular protozoon, is a long-lasting infectious disease characterized by weight loss, cough, fever, diarrhea, hepatosplenomegaly, and lethargy. Since not all symptoms appear simultaneously, and many other conditions manifest the same symptoms, the diagnosis is not always opportune. This disease is different from the anthroponotic form of visceral leishmaniasis found in Sudan and India. In the New World this disease is more common among poor, malnourished children (3) under 15 years of age who live in semiarid regions. The case-fatality rate is low if pentavalent antimony therapy is introduced promptly (4). The disease has also been reported in immunodepressed persons, especially those with HIV infection.

The domestic dog is the principal animal reservoir of *L. chagasi* and, as the constant companion of humans throughout the endemic-disease area, contributes to the dispersal of diseases during human migrations. The protozoon is transmitted from one mammalian host to another, including humans, primarily by the bite of a sand fly who has fed on an infected dog. In the Americas, the principal sand fly vector is *Lutzomyia longipalpis* (5). This 2- to 3-mm long fly has peridomestic and intradomiciliary habits (6) and avidly bites humans at night, primarily during twilight, while the host is resting.

Visceral leishmaniasis is usually diagnosed by identifying the parasite in spleen aspirates; microscope examination of bone marrow aspirates offers a satisfactory alternative. Specific antibodies may be detected by immune-enzymatic reactions and immunofluorescence, but these methods lack specificity and are too complex to be carried out in the field. TRALd, a recently developed diagnostic test, consists of a 60-second dipstick, based on a recombinant protein rK39 of a sequence of 298 amino acids and an improved serologic procedure (7). The procedure is undergoing field testing and appears to be a promising tool for control programs.

Although visceral leishmaniasis was previously known as a rural disease, large outbreaks and epidemics of visceral leishmaniasis have been reported recently in large cities in Brazil because of the favorable epidemiologic conditions associated with the reduction of the natural ecologic space of this zoonosis. Waves of droughts, lack of available farm land, and famine have led to a large migration of the population, to the peripheral suburbs of large cities, creating densely populated settlements of shanties (*favelas*) with minimal infrastructure and sanitation. Most families that migrate are young, less-established farmers of peak child-bearing age; children under 15 years of age account for a large percentage of the entire population. In these communities, the newly introduced disease (parasite) encounters a vast number of nonimmune hosts who, because of poor living conditions, are malnourished; malnutrition is one of the prime risk factors for *L. chagasi* infection and visceral leishmaniasis (3). The habit of keeping domestic animals such as dogs, chickens, and horses in the back yard provides an abundance of blood meals for sand fly vectors and raises vector population densities dramatically.

Two recent examples of reemerging visceral leishmaniasis in Brazil occurred in the cities of Teresina (population = 678,000), the capital of Piauí State, and São Luís (population = 918,000), the capital of Maranhão State. In Teresina, which has the best-documented records, an epidemic of visceral leishmaniasis occurred from 1981 to 1985; it was initially limited to rural settings, but later spread to peripheral areas of the city (9) (Figure 1). Spraying with residual insecticide helped bring the disease under control during the subsequent 3 years. Since 1989, visceral leishmaniasis has gradually reemerged; it reached epidemic levels by the end of 1992 and peaked in 1994. In the State of Maranhão, the disease reemerged in 1993,

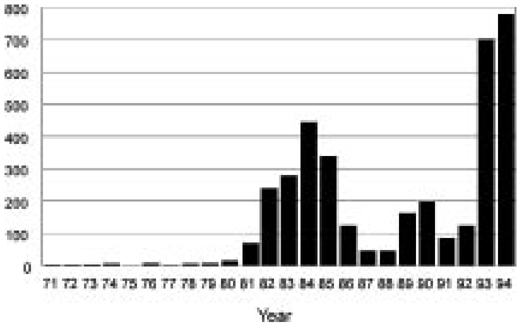

Figure 1. Number of cases of visceral leishmaniasis from the state of Piauí, Brazil, 1971 to 1994.

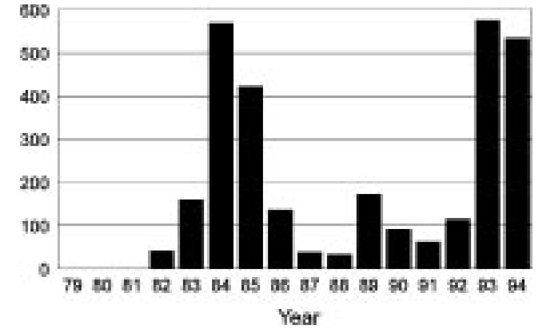

Figure 2. Number of cases of visceral leishmaniasis from the state of Maranhão, Brazil, 1979 to 1994.

(Figure 2), 10 years after a previous epidemic (1982 to 1986). In both Teresina and São Luís, the epidemics were preceded by prolonged, severe drought. The disease was found predominantly among young people who came from rural areas and lived in periurban, substandard housing and kept domestic animals in their back yards. The two states accounted for approximately 40% to 50% of the number of cases reported in the country during 1993 to 1994 (3,000/year).

Emergency control plans based on rapid diagnosis, complete treatment of human patients, spraying of residual insecticide, ultra-low volume spraying, elimination of infected (seropositive) dogs, and health education were initiated in these two cities in July 1994 (10). The incidence rate dropped markedly from January 1995, as expected, since the incubation period of visceral leishmaniasis is 4 to 6 months (11, 12). The remaining reported cases include those with longer incubation periods, as well as others, because of the lack of complete coverage of the control interventions and reduced sensitivity of the current diagnostic tools.

Technical expertise and effective mechanisms to prevent visceral leishmaniasis epidemics and urban transmission exist, but they require social and political commitment as well as the allocation of funds to provide adequate sanitary and satisfactory housing conditions to the population at risk. Visceral leishmaniasis in the Americas must be addressed before it becomes a serious public health problem. Even though it is a zoonosis, control interventions are available that, when properly used, can eliminate urban transmission and keep disease incidence in rural areas at a very low level.

**Jorge R. Arias, Ph.D.,* Pedro S. Monteiro,† and Fabio Zicker, M.D., Ph.D.‡**

*Pan American Health Organization, Brasilia, Brazil; †National Visceral Leishmaniasis Control Program, Ministry of Health, Brasilia, Brazil; ‡Pan American Health Organization, Washington, D.C.

### References

1. Pan American Health Organization/World Health Organization. Regional plan for action for combating new, emerging, and reemerging infectious diseases. Resolution CD38/17, August 1995. Pan American Health Organization, Washington, D.C., USA.
2. World Health Organization. Report of the Second WHO Meeting on Emerging Infectious Diseases. Document WHO/CDS/BVI/95.2. Geneva, Switzerland: World Health Organization, January 1995.
3. Cerf BJ, Jones TC, Badaro R, Sampaio D, Carvalho EM, Rocha H, Texeira R, Johnson Jr WD. Malnutrition as a risk factor for severe visceral leishmaniasis. J Infect Dis 1987;156:1030-2.
4. World Health Organization. Control of the leishmaniases: report of a WHO expert committee. Technical Report Series 793. Geneva, Switzerland: World Health Organization, 1990.
5. Lainson R, Shaw JJ. Epidemiology and ecology of leishmaniasis in Latin America. Nature 1978;273 (Parasit Suppl):595-600.
6. Young DG, Arias JR. Flebotomos: vectores de leishmaniasis en Las Americas. Pan American Health Organization, 1992: Cuaderno Técnico No. 33. Pan American Health Organization, Washington, D.C., USA.
7. Reed SG, Sheffler WG, Burns Jr JM, Scott JM, Orge MG, Ghalib HW, et al. An improved serodiagnostic procedure for visceral leishmaniasis. Am J Trop Med Hyg 1990;43:632-9.
8. Wilson ME. Travel and emergence of infectious diseases. Emerging Infectious Diseases 1995;1:39-49.
9. Costa CHN, Pereira HF, Araújo MV. Epidemia de leishmaniose visceral no estado do Piauí, Brasil, 1980-1986. Rev Saúde Públ S Paulo 1990;24:361-72.
10. Ministerio da Saúde. Controle, diagnóstico e tratamento da leishmaniose visceral (calazar). normas técnicas. Normas Técnicas. 1a. edição, Brasilia, Brazil: Fundação Nacional de Saúde, Brasil, 1994.
11. Manson-Bahr PEC, Southgate BA, Harvey AEC. Development of kala-azar in man after inoculation with a *Leishmania* from a Kenya sandfly. Br Med J 1963;I:1208-10.
12. Kirk R. Studies in leishmaniasis in Anglo-Egyptian Sudan. Trans R Soc Trop Med Hyg 1942;35:257-70.
